# Hyperoxia disrupts pulmonary epithelial barrier in newborn rats via the deterioration of occludin and ZO-1

**DOI:** 10.1186/1465-9921-13-36

**Published:** 2012-05-04

**Authors:** Kai You, Xuewen Xu, Jianhua Fu, Shuyan Xu, Xiaohong Yue, Zhiling Yu, Xindong Xue

**Affiliations:** 1 Department of Pediatrics, Shengjing Hospital of China Medical University, Shenyang, 110004, China; 2 Department of Urology, Shengjing Hospital of China Medical University, Shenyang, 110004, China; 3 Department of Pediatrics, Fengtian Hospital Affiliated to Shenyang Medical College, Shenyang, 110024, China

**Keywords:** Acute lung injury, Hyperoxia, Newborn, Permeability, Tight Junction

## Abstract

**Background:**

Prolonged exposure to hyperoxia in neonates can cause hyperoxic acute lung injury (HALI), which is characterized by increased pulmonary permeability and diffuse infiltration of various inflammatory cells. Disruption of the epithelial barrier may lead to altered pulmonary permeability and maintenance of barrier properties requires intact epithelial tight junctions (TJs). However, in neonatal animals, relatively little is known about how the TJ proteins are expressed in the pulmonary epithelium, including whether expression of TJ proteins is regulated in response to hyperoxia exposure. This study determines whether changes in tight junctions play an important role in disruption of the pulmonary epithelial barrier during hyperoxic acute lung injury.

**Methods:**

Newborn rats, randomly divided into two groups, were exposed to hyperoxia (95% oxygen) or normoxia for 1–7 days, and the severity of lung injury was assessed; location and expression of key tight junction protein occludin and ZO-1 were examined by immunofluorescence staining and immunobloting; messenger RNA in lung tissue was studied by RT-PCR; transmission electron microscopy study was performed for the detection of tight junction morphology.

**Results:**

We found that different durations of hyperoxia exposure caused different degrees of lung injury in newborn rats. Treatment with hyperoxia for prolonged duration contributed to more serious lung injury, which was characterized by increased wet-to-dry ratio, extravascular lung water content, and bronchoalveolar lavage fluid (BALF):serum FD4 ratio. Transmission electron microscopy study demonstrated that hyperoxia destroyed the structure of tight junctions and prolonged hyperoxia exposure, enhancing the structure destruction. The results were compatible with pathohistologic findings. We found that hyperoxia markedly disrupted the membrane localization and downregulated the cytoplasm expression of the key tight junction proteins occludin and ZO-1 in the alveolar epithelium by immunofluorescence. The changes of messenger RNA and protein expression of occludin and ZO-1 in lung tissue detected by RT-PCR and immunoblotting were consistent with the degree of lung injury.

**Conclusions:**

These data suggest that the disruption of the pulmonary epithelial barrier induced by hyperoxia is, at least in part, due to massive deterioration in the expression and localization of key TJ proteins.

## Background

Therapy with a high concentration of oxygen (hyperoxia) is often necessary to treat neonates with respiratory failure in neonatal intensive care units. However, one of the main complications is hyperoxia-induced lung injury, which may later result in serious sequela, such as bronchopulmonary dysplasia (BPD) [1,2]. The neonates with BPD continue to show decreased lung capacity at school age and even as young adolescents [3-5]. BPD has a negative impact not only on short-term and long-term pulmonary function but also on overall growth and neurodevelopment of these infants [6,7]. However, the mechanism of hyperoxia-induced lung injury is still not completely understood and currently there are no evidence-based effective strategies to prevent or treat this disease. Thus, there is an urgent need to understand how hyperoxia exposure initially influences pulmonary structure and function and how this lastingly disrupts lung development.

It is well known that the major pathologic finding associated with hyperoxia exposure at the early stage is hyperoxia-induced acute lung injury, which is frequently accompanied by an inflammatory response within lungs [8,9]. This response is characterized by increased pulmonary permeability and diffuse infiltration of various inflammatory cells [10-12]. Disruption of the epithelial barrier may lead to altered pulmonary permeability and airways fluid accumulation [13,14]. However, little attention has been done on the molecular mechanisms involved in disruption of the pulmonary epithelial barrier in neonatal hyperoxic lung injury.

The permeability barrier in terminal airspaces of the lung is due in large part to tight junctions between alveolar epithelial cells, which are continuous, circumferential, belt-like structures and regulate the flow of molecules between apical and basolateral compartments [15,16]. Tight junction is a primary barrier that regulates the diffusion of solutes through the paracellular pathway. Occludin and zonula occludens 1 (ZO-1) are two well characterized proteins in tight junctions. Occludin is a transmembrane protein which provides most of the barrier function of the tight junction [17,18]. Zonula occludens 1 is an intracellular protein, located between occludin and cytoskeletal proteins and thought to affect paracellular permeability [19]. Occludin and ZO-1 proteins form the intact intercellular barrier at tight junctions, and differences in the expression of these key TJ proteins account for differences in barrier function and paracellular permeability among epithelia [20-23]. For example, previous studies in intestinal epithelia have shown that serious impairment of intestinal barrier function is associated with an increased permeability and morphological changes through downregulation and localization shift of occluding [24,25]. Others have reported that changes in occludin and ZO-1 expression in response to environmental stimuli may account for changes in barrier properties in the pulmonary epithelia [24,26-28].

However, our current understanding of the structural components and regulation of tight junctions in the alveolar epithelium is insufficient, and previous studies were mainly performed in adult animals or cells and have scarcely been examined in the neonatal period. The primary objective of this study was to determine whether there were changes in alveolar barrier structure and function in newborn rats exposed to hyperoxia and whether tight junction proteins were differentially expressed in the lung during experimental acute lung injury induced by hyperoxia and whether these changes account for impairment in alveolar barrier properties and are relevant to pulmonary edema formation.

## Methods

### Animals and hyperoxia exposure protocol

Time-dated, pregnant Wistar rats (200–220 g) were purchased from the Center for Experimental Animals of China Medical University. All animal procedures were reviewed and approved by the Laboratory Animal Ethics Committee of China Medical University. Pups were delivered naturally at term gestation (22 days). The full-term newborn rats from twelve litters were randomly marked and assigned to two groups and were exposed to hyperoxia (95% oxygen; experimental group) or normoxia (21%; control group) beginning on the day of birth. The inhaled oxygen concentration was measured and recorded continuously with an analyzer equipped with a strip-chart recorder (model 572; Servomex, Norwood, MA). Humidity was routinely 60%–70%. Nursing rat dams were switched every 24 hours between the hyperoxic and normoxic chambers, to avoid oxygen toxicity and provide equal nutrition to each litter. Chambers were open for 10 min per day for weighing and cage cleaning. Pups were sacrificed and lungs were harvested at the end of 1, 3, 5 and 7 days of exposure.

### Lung histology

The lungs of a random sample from each group were inflated by intratracheal instillation of 4% formaldehyde solution at a hydrostatic pressure of 18 cmH_2_O. The lungs were then fixed in 4% paraformaldehyde for 24 h. Lung tissue was dehydrated with graded alcohol, placed in xylene for 1 h and then embedded in paraffin at 60°C. Sections of lung (5 μm) were stained with hematoxylin and eosin. Lung sections from all lungs were examined for any histological changes. From each section, 20 random areas were examined at × 400 magnification. Within each field, lung injury was scored according to a new histologic acute lung injury (ALI) scoring system [29]. Histopathological evaluation was performed by an independent pathologist who was blinded to the experimental group.

### Wet/dry lung weight ratio and excess lung water measurement

The right upper lung lobes from random samples of two groups were excised at the end of the experiment and were weighed to determine the wet lung weight. After that, the tissues were placed in an oven and maintained at a temperature of 80°C for 48 hours before being weighed again to give final dry weight. The presence of pulmonary edema was examined by calculating the pulmonary wet/dry ratio (W/D).

To determine the extravascular water in the lung, a blood sample was obtained from cardiac puncture to measure the hemoglobin concentration. After the determination of lung wet weight, the tissue was homogenized and dried. Measurements of the hemoglobin concentration in the lung homogenate also allowed for the calculation of excess lung water according to the standard methods used as previously described [30,31].

### Measurement of bronchoalveolar epithelial permeability

Pups were injected via the saphenous vein with fluorescein isothiocyanate-conjugated dextran 4000 (FD4) solution in PBS (25 mg/ml; 10 mg/kg) after the anesthesia. Fifteen minutes later, the trachea was exposed, and the lungs were lavaged three times with 1 ml of sterile saline per wash. The aliquots of bronchoalveolar lavage fluid (BALF) were collected. Blood was obtained by cardiac puncture, and the serum was collected by centrifugation at 3,000 rpm for 5 min. The concentrations of FD4 in the BALF and serum (20 fold diluted by PBS) were determined with a spectrofluorometer (F-4500; Hitachi, Tokyo, Japan) using an excitation filter of 492 nm and an emission wavelength of 515 nm. The BALF:serum fluorescence ratio was calculated and used as a measure of pulmonary epithelial permeability.

### Transmission electron microscopy study

After dissection of lung, the specimens were fixed in 2.5% glutaraldehyde overnight at 4°C. The tissues were washed in phosphate buffer (pH 7.4), postfixed in 1% osmium tetroxide in phosphate buffer (pH 7.4), dehydrated in graded ethanol solutions, treated in propylene oxide and embedded in epoxy-resin embedding media. 60-nm thin transverse random sections were collected on single copper slot grids coated with parlodion, stained with uranyl acetate and lead citrate, and observed with a JEM-1200EX transmission electron microscope (Hitachi Electronic Company, Japan). Images were documented by using Kodak SO163 EM film.

### Immunofluorescence staining

Lungs of right middle lobe were inflated with 4% paraformaldehyde, soaked in 3% paraformaldehyde for 3 h at 4°C, cryoprotected in 30% sucrose for 12 h at 4°C and frozen at 80°C. Frozen sections (10 μm) were air dried and then washed three times with PBS. The sections were incubated with 0.5% Triton X-100 for 5 min at room temperature and then washed three times with PBS. The sections were then blocked with 10% goat serum for 30 min at 37°C and incubated with primary antibodies as follows: 1:200 diluted occludin (mouse monoclone antibody; Zymed Laboratories, South San Francisco, Calif) and 1:80 diluted ZO-1 (rabbit polyclone antibody; Zymed Laboratories, South San Francisco, Calif) together overnight at 4°C. As negative controls, some sections were incubated in the absence of the primary antibodies. Tissue sections were washed four times with PBS-Triton. The second antibody against mouse was conjugated with Alexa fluor-488 (green fluorescence) and the anti-rabbit antibody with Alexa fluor-594 (red fluorescence) for 60 min at 37°C. Sections were washed three times with PBS, and the nuclei were stained with DAPI (1:2000; Sigma Chemical) for 2 min. After sufficient washes, images were captured by confocal laser scanning microscopy (MTC-600, BIO-RAD, USA).

### Immunoblotting

Briefly, equal amounts of protein extract were mixed in 1 × Laemmli buffer and boiled for 3 min. The proteins were electrophoresed on 7.5% precast SDS-polyacrylamide gels (80 V for 120 min). Then the proteins were transferred electrophoretically onto polyvinylidene difluoride (PVDF) membranes (40 V for 180 min). The membranes were incubated for 1 h in 1:10 normal donkey serum to block nonspecific binding. The PVDF membranes were then incubated overnight at 4°C with primary antibodies occludin (1:500), ZO-1 (1:250), and β-actin (1:4,000) diluted in PBS-0.02% Tween 20. Some samples were incubated in the solution without primary antibodies to be negative controls. After washing three times in PBS, the membranes were incubated at room temperature for 90 min with horseradish peroxidase-conjugated secondary antibody. The membrane was then washesd in PBS and impregnated with the enhanced chemiluminescence substrate (ECL kit; Santa Cruz Biotechnology) and was used to expose the radiograhic film. All the bands were scanned with ChemiImager 5500 V2.03 software, and IDVs were calculated by computerized image analysis system (Fluor Chen 2.0) and normalized to that of β-actin.

### RT-PCR

Differential expression of occludin and ZO-1 was validated by a separate comparison of new subjects using RT-PCR. These studies compared spontaneously pups in normoxia group with pups exposured to hyperoxia. Briefly, lung tissue was dissected and homogenized. RNA was extracted (Invitrogen, Camarillo, Calif, USA) following the manufacturer's instructions. cDNA was generated from 1 μg total of each sample (SuperScript III; Invitrogen). The sequences of following primers were designed by Primer Premier 5.0 software (Premier Biosoft International, Palo Alto, Calif) and are shown in Table 1. The amplification reaction was carried out in 30 cycles at 95°C for 45 s, 55°C for 45 s, and 75°C for 1 min in a thermal cycler. PCR products were electrophoresed on 2.5% agarose gels. Gels were photographed by a ChemiImager 5500 gel image analysis instrument (Alpha Innotech, USA). The integrated density values (IDVs) of PCR product bands were calculated by computerized image analysis system (Fluor Chen 2.0) and expressed relative to that of β-actin.

**Table 1:** List of primers used for measuring gene expression by RT-PCR

**Gene name**	**Forward Primer**	**Reverse Primer**
Occludin	TTGGGAGCCTTGACATCTTGTTC	GCCATACATGTCATTGCTTGGTG
ZO-1	CCATCTTTGGACCGATTGCTG	TAATGCCCGAGCTCCGATG
β-actin	GGAGATTACTGCCCTGGCTCCTA	GACTCATCGTACTCCTGCTTGCTG

### Statistical analysis

SPSS 18.0 software was used for this statistical analysis. Data were summarized as mean ± SD. Student *t*-test was used to determine the significant difference between two groups. One-way analysis of variance (ANOVA) and post hoc comparisons (Bonferroni test) were used to determine the significant difference among multiple groups. A P value of less than 0.05 was considered to be statistically significant.

## Results

### Lung histology shows levels of lung injury after different days of hyperoxia exposure

**Figure 1:**
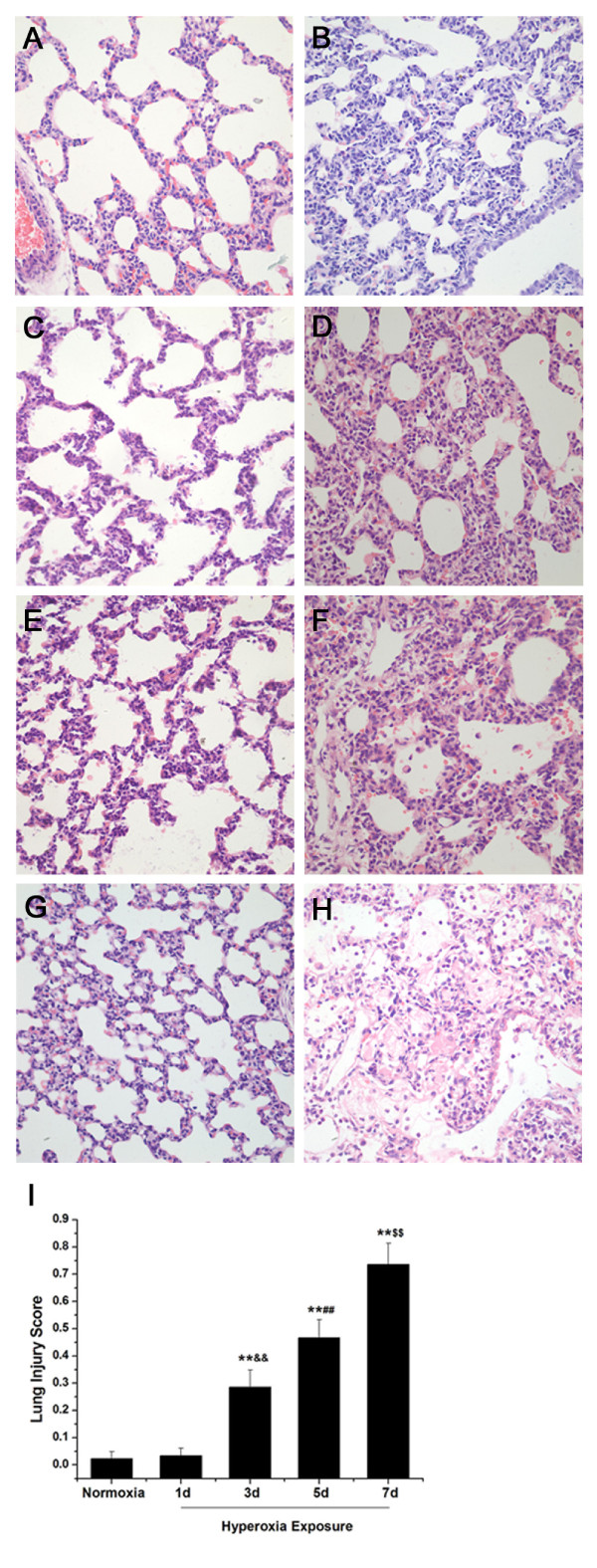
** Lung histology shows levels of lung injury after different days of hyperoxia exposure (original magnification × 400).** The alveolar-like structures in full-term newborn rat lungs exposed to normoxia were irregular, and there were a small number of septa on the first day (**A**). There was no obvious difference in pulmonary morphology in newborn rats exposed to hyperoxia compared with that of the normoxia group on day 1 (**B**). The septa became thinner and the numbers increased in normoxia group on the third day (**C**). The septa were thickened and there was neutrophils infiltration in the interstitial space in hyperoxia group on day 3 (**D**). On the fifth day, the alveoli grew in number, and the septa continued to be thinner in normoxia group (**E**). In the hyperoxia group, there was more severe interstitial edema and a few inflammatory cells infiltration in the alveolar space on day 5 (**F**). The alveoli were regular and uniformly distributed in newborn rats exposed to normoxia on the seventh day (**G**). In the hyperoxia group, there was more inflammatory cells and red blood cell infiltration in the alveolar space with proteinaceous debris filling the airspaces on day 7 (**H**). Lung injury scores following exposure of newborn rats to normoxia for 1d or hyperoxia for 1d, 3d, 5d and 7d. 20 random high-power fields (400 × total magnification) were independently scored in a blinded fashion for each condition (**I**). Data are the mean ± SEM (n = 6 in each group); ** *P* < 0.01 versus normoxia group; **&&***P* < 0.01 versus 1 day of hyperoxia exposure group; **##***P* < 0.01 versus 3 days of hyperoxia exposure group; **$$***P* < 0.01 versus 5 days of hyperoxia exposure group.

Formalin-fixed paraffin-embedded lung tissues from newborn rats after different days of hyperoxia or normoxia exposure were treated with hematoxylin-eosin (HE) staining. On the first day, the alveolar-like structures in full-term newborn rat lungs exposed to normoxia were irregular, and a small number of septa could be seen (Figure 1A). There was no obvious difference in pulmonary morphology in newborn rats exposed to hyperoxia compared with that of the normoxia group on day 1 (Figure 1B). The septa became thinner and the numbers increased in the normoxia group on the third day (Figure 1C). The septa were thickened and vascular congestion was also observed. There was neutrophil infiltration in the interstitial space in the hyperoxia group on day 3 (Figure 1D). On the fifth day, the alveoli grew in number, and the septa continued to be thinner in the normoxia group (Figure 1E). After 5 days of hyperoxia, histological evaluation of lung tissue in newborn rats revealed increased neutrophil infiltration in the interstitium. There was more severe interstitial edema and a few inflammatory cells infiltrating the alveolar space (Figure 1F). The alveoli were regular and uniformly distributed in newborn rats exposed to normoxia on the seventh day (Figure 1G). In the hyperoxia group, there was increased loss of pulmonary epithelial architecture, more inflammatory cells and red blood cell infiltration in the alveolar space with proteinaceous debris filling the airspaces on day 7 (Figure 1H). After 7 days of hyperoxia, the lung injury appeared more severe than that observed on day 3 or day 5. Treatment with hyperoxia for 3 days resulted in a less severe injury to lung tissue compared with that observed on day 5. The observation demonstrated that more severe lung injury happened in newborn rats with longer hyperoxia exposure. Lung injury scores, shown in Figure 1I, supported the observation that the lung injury level of newborn rats treated with hyperoxia was time-dependent.

### Effect of hyperoxia exposure on pulmonary edema

**Figure 2:**
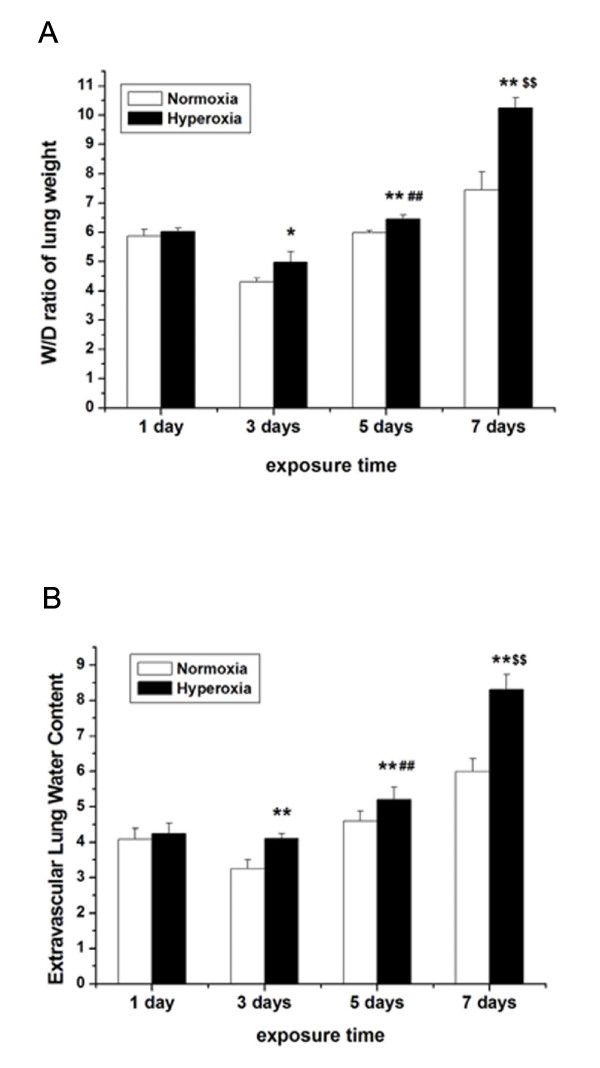
** Effect of hyperoxia exposure on pulmonary edema after different days of hyperoxia exposure.** Effects of hyperoxia exposure on pulmonary edema were determined by the wet-to-dry (W/D) ratio of lung weight (**A**) and extravascular lung water content (**B**) following exposure of newborn rats to normoxia or hyperoxia for 1, 3, 5 and 7 days. Hyperoxia significantly increased the W/D ratio of lung weight and extravascular lung water content compared with normoxia on 5 and 7 days. Data are the mean ± SEM (n = 6 in each group); *P < 0.05, ** *P* < 0.01 versus normoxia group; **##***P* < 0.01 versus 3 days of hyperoxia exposure group; **$$***P* < 0.01 versus 5 days of hyperoxia exposure group.

Pulmonary edema formation, indicative of injury to the pulmonary epithelial barrier, often happens with an increase in the total water W/D ratio. Our study revealed there was no difference in W/D ratio between the normoxia group and the hyperoxia group on the first day (*P* > 0.05). On the third day, W/D ratio in the hyperoxia groups was significantly higher than that of the normoxia group (*P* < 0.05). After 5 days of hyperoxia exposure, W/D ratio was much higher than that of the normoxia group (*P* < 0.01). There was significant increase in W/D ratio in newborn rats with 7 days of hyperoxia exposure compared with that in corresponding normoxia group (*P* < 0.01) (Figure 2A).

The measurement of extravascular lung water content is also valuable in studies of mechanisms of increased permeability pulmonary edema. The extravascular lung water content in newborn rats with hyperoxia exposure was higher than that with normoxia exposure and the results were almost the same with the lung W/D ratio (Figure 2B).

### Hyperoxia exposure decreases pulmonary epithelial barrier function

**Figure 3:**
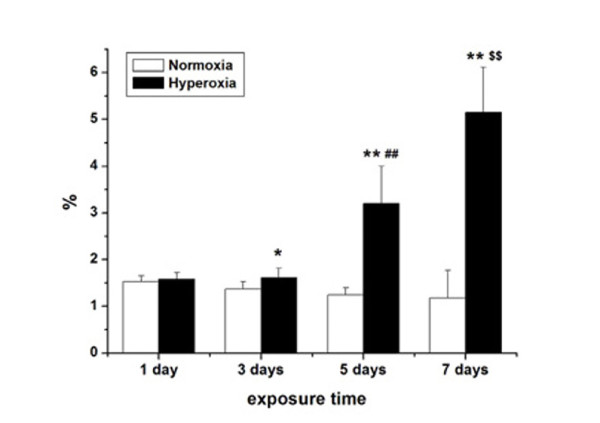
** The permeability of pulmonary epithelia after different days of hyperoxia exposure.** Newborn rats received an intravenous injection of FD4. Fifteen minutes later, bronchoalveolar lavage fluid (BALF) and serum were collected, and the BALF:serum FD4 ratio was determined. Data are the mean ± SEM (n = 6 in each group); *P < 0.05, ** *P* < 0.01 versus normoxia group; **##***P* < 0.01 versus 3 days of hyperoxia exposure group; **$$***P* < 0.01 versus 5 days of hyperoxia exposure group.

Generally, pulmonary epithelial barrier function decreases with an increase of bronchoalveolar epithelial permeability. We monitored the changes in pulmonary epithelial barrier function by examining the effect of hyperoxia exposure on bronchoalveolar epithelial permeability. Bronchoalveolar epithelial permeability was determined by measuring the leakage of FD4 from serum into BALF in rats (Figure 3). On the first day, there was no obvious difference in the BALF:serum fluorescence ratios between the hyperoxia and normoxia groups (*P* > 0.05). After 3 days of hyperoxia exposure, the ratios of BALF:serum fluorescence in newborn rats were significantly higher than those in normoxia group (*P* < 0.05). The BALF:serum fluorescence ratios in hyperoxia group were approximately 2-fold higher than those in the control group on day 5 (*P* < 0.01). On the seventh days, the BALF:serum FD4 ratios in hyperoxia group further increased and were more than 3-fold higher than those in the normoxia group (*P* < 0.01). Prolonged treatment with hyperoxia exposure significantly increased the bronchoalveolar epithelial permeability.

### Morphological alterations of tight junction in alveolar epithelia during hyperoxia exposure

**Figure 4:**
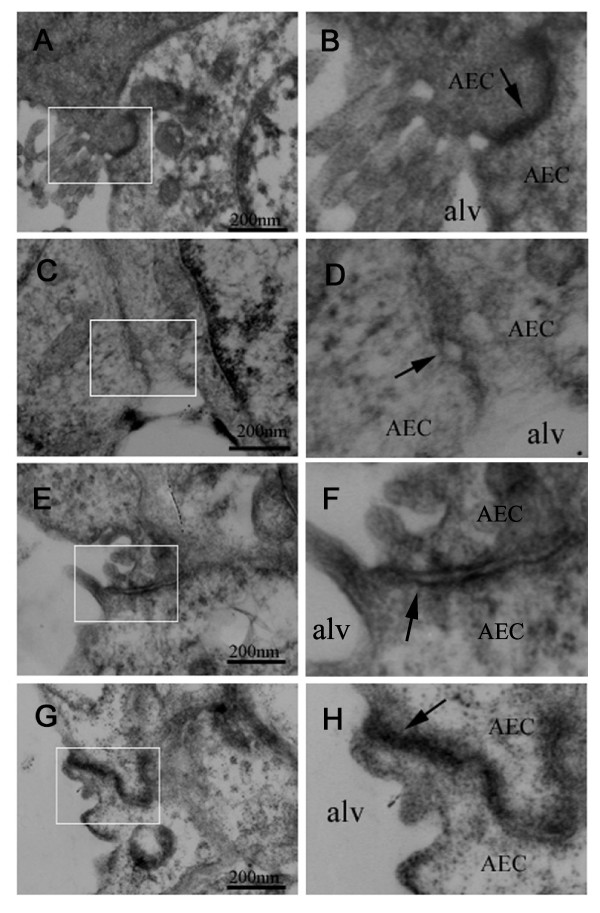
** Hyperoxia exposure disrupts alveolar epithelial tight junctions.** The integrity of pulmonary epithelium and morphological alterations of tight junction were studied by transmission electron microscopy in both normoxia and hyperoxia groups. Tight junctions between pulmonary epithelial cells were extremely tight in the lungs from normoxia-treated newborn rats (**A** and **B**). After treatment with hyperoxia for 7 days, tight junctions were irregularly widened (**C** and **D**). Tight junctions were open between epithelial cells in newborn rats with hyperoxia for 5 days, and paracellular gaps were visible between alveolar epithelial cells (**E **and **F**). Intact tight junctions remained between alveolar epithelial cells in newborn rats exposed to hyperoxia for 1 and 3 days (**G **and **H**).

Since hyperoxia exposure increased the bronchoalveolar epithelial permeability in newborn rats, it might alter the morphology of tight junction in the alveolar epithelium. The integrity of pulmonary epithelium and morphological alterations of tight junction were studied by transmission electron microscopy in newborn rats with normoxia and hyperoxia exposure. Random sampling of left lung from newborn rats exposed to normoxia showed obvious and frequent junctions between all epithelial cells which were extremely tight (Figure 4A). The tight junctions were ultrastructurally normal (Figure 4B). In contrast, after treatment with hyperoxia for 7 days, random sampling of left lungs from newborn rats showed loss of tight junctions between alveolar epithelial cells (Figure 4C). The tight junctions were irregularly widened (Figure 4D). Epithelial cells appeared retracted from each other with clear paracellular gaps. After 5 days of hyperoxia exposure, the tight junctions were also widened but to a much less extent (Figure 4E, F). Morphology of tight junction showed no observable changes in newborn rats exposed to hyperoxia for 1 day or 3 day (Figure 4G, H).

### Effects of hyperoxia exposure on the localization and expression of ZO-1 and occludin in alveolar epithelia

**Figure 5:**
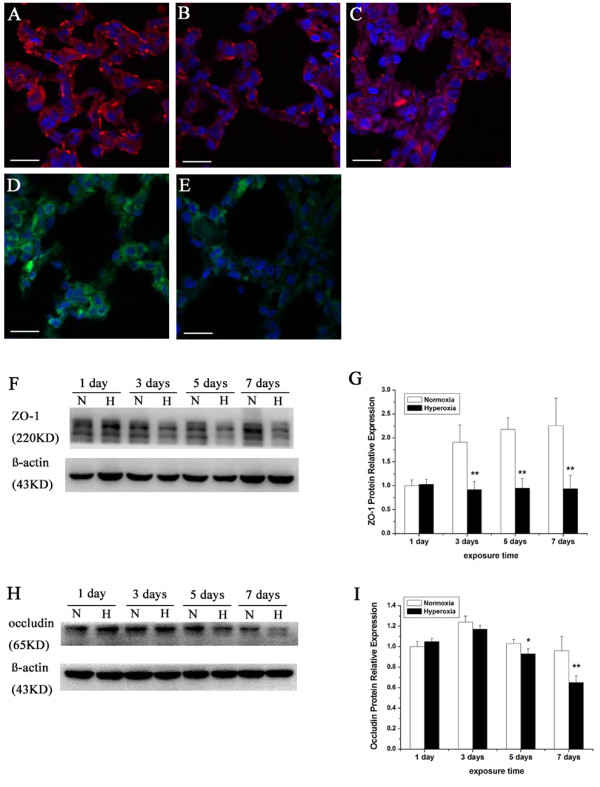
** Hyperoxia exposure alters location and expression of ZO-1 and occludin in alveolar epithelial.** Immunostaining and western blot analysis were performed to identify alteration in location and expression of ZO-1 and occludin in alveolar epithelial. A continuous line along the membrane and diffuse in the cytoplasm staining of ZO-1 and occludin was shown in the alveolar epithelium of control rats (A, D). After 3 and 5 days, hyperoxia exposure induced partial breakdown of membrane staining and decreased cytoplasm staining of ZO-1 in the alveolar epithelium (B). The staining intensity of ZO-1 and occludin was dramatically reduced in lung tissues from rats which were exposed to hyperoxia for 7 days (C, E). Bars represent 50 μm. After 7 days of hyperoxia exposure, the expression of ZO-1 and occludin dramatically declined and was virtually undetectable examined by Western blotting. β-actin immunoreactive bands were used to demonstrate equal loading (F, H). Densitometric quantification of ZO-1 and occludin protein levels was expressed as fold increase compared with normoxia for 1 day group (set as 1) (G, I). Values are presented as means ± SD; *P < 0.05, ** *P* < 0.01 versus normoxia group. The results shown are representative of experiments that were repeated 4 times.

The morphology of tight junctions in the alveolar epithelium altered when exposed to hyperoxia. Then the localization and expression of tight junction proteins in the alveolar epithelium might change. ZO-1 and occludin, which are two of the best-characterized tight junction proteins, were examined by immunofluorescence staining from frozen sections of the right middle lobe. ZO-1 was localized in lung tissue from rats with normoxia exposure as a continuous line along the boundaries between neighboring bronchial and alveolar epithelial cells (Figure 5A). Within one day of hyperoxia exposure, ZO-1 distribution in tight junctions was not obviously diminished. After 3 and 5 days, hyperoxia exposure induced partial breakdown of membrane staining and decreased cytoplasm staining of ZO-1 in the alveolar epithelium (Figure 5B). Treatment with hyperoxia for 7 days induced a fragmented staining pattern on the membrane (Figure 5C). The features of occludin immunostaining were similar to that of ZO-1. A consecutive line along the membrane and diffuse in the cytoplasm staining of occludin was shown in the alveolar epithelium of control rats (Figure 5D). The intensity of this staining was dramatically reduced in lung tissues from rats which were exposed to hyperoxia for 7 days (Figure 5E).

Furthermore, we detected the protein expression of ZO-1 and occludin from lung tissue by immunoblotting. Hyperoxia exposure can induce different levels of tight junction proteins expression in neonatal rat lung tissue after 1–7 days of duration. The protein expression levels of ZO-1 increased slightly after one day of hyperoxia exposure and clear ZO-1 bands were apparent in both experimental and control group on day 1. However, the decrease of ZO-1 expression was statistically significant after 3 days of hyperoxia exposure (*P* < 0.01). Further decreased expression of these proteins was observed in newborn rats with prolonged hyperoxia exposure (Figure 5F, G). The decrease of occludin expression was statistically significant on day 5 (*P* < 0.05). After 7 days of hyperoxia exposure, the expression dramatically declined and was virtually undetectable by Western blotting (Figure 5H, I).

### Hyperoxia exposure decreases the mRNA expression of ZO-1 and occludin in the lung of newborn rats

**Figure 6:**
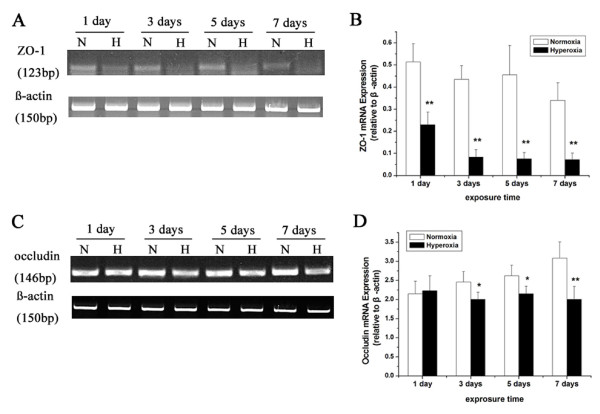
** mRNA expression levels of ZO-1 and occludin in lung tissue.** RNA was extracted from the lung tissue in both normoxia and hyperoxia groups, converted to cDNA and quantification of ZO-1 and occludin mRNA expression was determined using RT-PCR. β-actin was used as an internal control (**A**, **C**). Semiquantitative analysis of ZO-1 and occludin after different days of hyperoxia exposure (**B**, **D**). Values are presented as means ± SD; * *P* < 0.05, ** *P* < 0.01 versus normoxia group. All experiments were performed independently 4 times.

The messenger RNA expressions of ZO-1 and occludin were further examined in lung tissue by RT-PCR. The results showed that 1 day of hyperoxia exposure significantly depressed the messenger RNA levels of ZO-1 (*P* < 0.01). The messenger RNA levels were markedly downregulated in newborn rats with prolonged hyperoxia exposure (*P* < 0.01) (Figure 6A, B). The decrease of occludin mRNA level was statistically significant after 3 or 5 days of hyperoxia exposure (*P* < 0.05). Treatment with hyperoxia for 7 days had the greatest depressive effect on these messenger RNA levels (*P* < 0.01) (Figure 6C, D). The results were consistent with the changes of these protein expressions in lung tissue.

## Disscussion

Although hyperoxia exposure is the most common cause of lung injury for neonates requiring respiratory support, the molecular and cellular mechanisms underlying this phenomenon remain elusive. Non-cardiogenic pulmonary edema and diffuse infiltration of inflammatory cells have been confirmed as the major pathologic findings in hyperoxia-induced acute lung injury [9,10]. Previous studies on mechanisms related to these pathologic changes are mostly focused on abnormal secretion of cytokines, and imbalance between oxidation and antioxidation [2,8,32,33]. However, treatment against those factors is insufficient [34-36]. Furthermore, the molecular mechanisms involved in disruption of the pulmonary epithelial barrier itself are seldom investigated in neonatal hyperoxic lung injury and no such research is concerned with how tight junctions change during the disease. Thus, our study was undertaken to explore possible mechanisms of impairment of pulmonary epithelial integrity and find what role tight junctions play in newborn animals with hyperoxia exposure.

Our study, for the first time, demonstrated alterations in the structure and function of tight junctions in hyperoxia-induced lung injury in newborn animals. We observed that hyperoxia exposure impaired pulmonary epithelial tight junctions with increased pulmonary epithelial permeability in an in vivo model. Also, we found that the expression of occludin and ZO-1 decreased during hyperoxia exposure and the alterations were consistent with changes in the barrier function of the bronchoalveolar epithelium, suggesting these key tight junction proteins might play important roles in the disruption of epithelial integrity in hyperoxic lung injury in newborn rats.

This study provided new evidence that hyperoxia exposure led to inflammation and pulmonary edema. According to the histologic findings, we confirmed that proteinaceous fluid and neutrophil infiltration in lung tissue markedly increased after hyperoxia exposure. Pulmonary neutrophil sequestration and transmigration are demonstrated as typical manifestations of acute lung injury [37,38]. In this study, the results of lung W/D ratio and extravascular lung water content reached a common consensus that, there was obvious pulmonary edema in newborn rats with prolonged hyperoxia exposure, which were comparable with the severity of changes in pathology. These results are in accordance with previous studies which show polymorphonuclear cell infiltration and edema formation in hyperoxia-induced ALI [39,40].

We also showed that hyperoxia exposure in newborn rats significantly increased bronchoalveolar epithelial permeability (about 2–4 fold). The magnitude of the functional alteration in pulmonary epithelial barrier peaked after 7 days of hyperoxia exposure while the BALF:serum FD4 ratio was almost unchanged on day 1. The data demonstrated treatment with hyperoxia for prolonged duration contributed to more serious lung injury. Our findings are consitent with other studies which show hyperoxia increases pulmonary permeability [41,42]. It has been determined that severe lung injury and inflammatory reaction was induced by 95% O2 for 3 and 7 days with protein leak across the alveolar epithelium increasing in rat pups [43]. BALF albumin levels and neutrophil count are significantly increased in adult rats with hyperoxia exposure, suggesting the alteration in alveolar epithelial barrier function [44].

Since maintenance of normal pulmonary barrier function depends on intact epithelial tight junctions [24,26], transmission electron microscopy was used in this study to examine their morphological alterations. We observed that treatment with hyperoxia in newborn rats destroyed the structure of tight junctions. Using immunofluorescence, we showed that hyperoxia exposure markedly disrupted the membrane localization and downregulated the cytoplasm expression of the key tight junction proteins occludin and ZO-1 in the alveolar epithelium and the results were confirmed by immunoblotting and RT-PCR. Functional opening of tight junction barrier and down-regulation of occludin and ZO-1 protein expression have been also found in acute lung injury in adult animals [27,28]. Previous studies in intestinal epithelia have shown that morphology of tight junctions change through downregulation and localization shift of occluding [24,25].

Furthermore, our study showed that prolonged hyperoxia exposure enhanced the structure destruction of epithelial tight junctions while decreasing in occludin and ZO-1 expression was more obvious. This temporal pattern of changes in the expression of key tight junction proteins was paralleled by changes in the barrier function of the bronchoalveolar epithelium, which suggested decreased synthesis of occludin and ZO-1 at a transcriptional level may be a mechanism for the rapid disruption of epithelial integrity in newborn animals with hyperoxia-induced lung injury. However, abnormal structure of the tight junction was not found in groups with 1 or 3 days of hyperoxia exposure. Also, pathohistologic findings showed there was no inflammation and protein leakage until the third day of hyperoxia exposure. This phenomenon suggests that mild-to-moderate deterioration of these proteins may not be enough to change the morphology of tight junctions and cause severe lung edema.

A preliminary study has given some clue that oxidative stress induces tyrosine phosphorylation and dissociates the occludin-ZO-1 complexes from the cytoskeleton by a tyrosine kinase-dependent mechanism [45]. Whether hyperoxia disrupts alveolar epithelial barrier function by means of tyrosine phosphorylation and dissociation of occludin-ZO-1 complexes needs further study.

## Conclusion

Taken together, this study demonstrates that hyperoxia exposure in newborn rats impairs the structure and function of alveolar epithelial tight junctions and the changes are enhanced with prolonged hyperoxia exposure. Our data demonstrate occludin and ZO-1 expressions decrease during hyperoxia-induced acute lung injury in neonatal animals and the alterations are consistent with the severity of lung injury. Therefore, we propose that the disruption of the pulmonary epithelial barrier induced by hyperoxia is, at least in part, due to massive deterioration in the expression and localization of key TJ proteins. How deterioration of these proteins impairs the function of pulmonary epithelial barrier needs further study.

## Abbreviations

BALF: Bronchoalveolar lavage fluid; BPD: Bronchopulmonary dysplasia; FD4: Fluorescein isothiocyanate-conjugated dextran 4000; HALI: Hyperoxic acute lung injury; TJ: Tight junction; ZO-1: Zonula occludens 1.

## Competing interests

The authors declare that they have no competing interests.

## Authors’ contributions

KY performed all the experiments, analyzed the data, and wrote the manuscript. JF and XXue participated in the design of the experiments and manuscript revision. XXu and SX participated in the animal studies and coordinated the immunostaining studies. ZY and XY helped to perform the statistical analysis. All authors read and approved the final manuscript.
